# Osteoporosis Treatments Affect Bone Matrix Maturation in a Rat Model of Induced Cortical Remodeling

**DOI:** 10.1002/jbm4.10344

**Published:** 2020-02-20

**Authors:** Ryan D Ross, Kyle Anderson, Reid Davison, Bilal M El‐Masri, Christina M Andreasen, Thomas L Andersen, Dale R Sumner

**Affiliations:** ^1^ Department of Cell & Molecular Medicine Rush University Medical Center Chicago IL USA; ^2^ Department of Orthopedic Surgery Rush University Medical Center Chicago IL USA; ^3^ Clinical Cell Biology, Research Unit of Pathology, Department of Clinical Research University of Southern Denmark, and Department of Pathology, Odense University Hospital Odense Denmark; ^4^ Department of Molecular Medicine University of Southern Denmark Odense Denmark

**Keywords:** BISPHOSPHONATE, BONE REMODELING, INTRACORTICAL, LACTATION, MINERALIZATION, SCLEROSTIN

## Abstract

To test how osteoporosis drugs affect bone matrix maturation during cortical bone remodeling, 72 pregnant rats were switched from a 0.4% to a 0.01% calcium diet at parturition for a 23‐day lactation period. At weaning, eight dams were sacrificed to establish baseline values, while the remaining dams were returned to 0.4% calcium and treated with vehicle (saline), sodium fluoride (NaF), zoledronic acid (ZA), or sclerostin antibody (Scl‐Ab) for either 7 or 28 days (eight animals per group per time point). Femora were examined by μCT, dynamic histomorphometry, Fourier transform infrared imaging, and three‐point bending of notched specimens. Cortical porosity decreased in all groups from baseline to day 28. Intracortical mineralizing surface (MS/BS) and mineral apposition rate (MAR), as well as the mineral‐to‐matrix ratio were unaffected by treatment, but intracortical crystallinity was increased in the ZA group at day 10 compared with vehicle. Cortical area increased in all groups over 28 days mainly because of an addition of bone at the endocortical surface. Endocortical MS/BS did not vary among the groups, but endocortical MAR was suppressed in the NaF group at day 2 and elevated in the Scl‐Ab group at day 4 compared with vehicle. Endocortical mineral‐to‐matrix ratio was increased at days 5 and 10 following NaF treatment and endocortical crystallinity was increased at day 5 following ZA treatment compared with vehicle. Fracture toughness did not differ among the groups. Thus, the treatments affected matrix maturation more strongly at the endocortical then intracortical envelope. In this model of induced remodeling, the bone formation phase is synchronized at multiple sites, facilitating study of the effects of drugs or other bone‐targeting agents on matrix maturation independent of their effects on the initiation of remodeling. © 2020 The Authors. *JBMR Plus* published by Wiley Periodicals, Inc. on behalf of American Society for Bone and Mineral Research.

## Introduction

Bone remodeling is a complex process that is initiated when osteoclasts resorb bone matrix and is completed only after the deposited collagen matrix is mineralized. Matrix maturation, or the process by which collagen matrix accumulates calcium phosphate mineral, provides bone tissue with its characteristic strength and stiffness. Nevertheless, the process of matrix maturation in the adult skeleton remains poorly understood, partly because a limited quantity of matrix is actively mineralizing at any given time and place. Using small animal models, one can induce remodeling and synchronize the formation phase making the process of matrix mineralization in the adult skeleton more amenable to study. To this end, our lab has shown in adult rats that the bone formation phase is synchronized at multiple remodeling sites following a regimen in which a low calcium diet during lactation is followed by return to normal calcium intake upon weaning.^1^, ^2^ The model is characterized by normal matrix mineralization kinetics^2^ and minimal effects on mineral metabolism during the formation period.^1^

Osteoporosis drugs alter bone mass by either inhibiting bone resorption or increasing bone formation.^3^, ^4^ Although the effects of these treatments on resorption and formation are generally well‐established by preclinical studies and clinical trials in which bone biopsies are analyzed, it is less common for researchers to investigate treatment effects on matrix maturation and the quality of the resulting matrix. The example of sodium fluoride (NaF) clearly demonstrates an instance where increasing bone mass while altering maturation can negatively affect drug efficacy. NaF was a promising osteoporosis treatment because it increased BMD.^5^ However, it became evident that the treated patients were at increased risk of fracture,^6^, ^7^ which was later attributed to NaF‐induced aberrant mineralization.^8^ Had there been a preclinical model to assess the effect of NaF on maturation, it is likely that these fractures could have been avoided.

The potential effects of current osteoporosis drugs on matrix maturation are not well‐studied. Thus, the precise nature of these effects and whether they negatively impact mechanical function remain unclear. Bisphosphonates, the most commonly prescribed class of bone‐targeting drugs, have been shown to regulate the expression of mineralization‐related genes in vitro; however, it is difficult to assess their effects on maturation in vivo because of the inhibited bone remodeling following treatment. Without resorption, there are very few sites of active tissue maturation in short‐term animal studies. However, in long‐term human biopsies, there have been reports of altered matrix mineralization following bisphosphonate treatment.^9^, ^10^, ^11^ Sclerostin protein has been reported to inhibit mineral accumulation in vitro,^12^, ^13^ which is consistent with in vivo evidence showing that suppression of sclerostin activity via monoclonal antibodies reduces the mineralization lag time^13^ and accelerates mineralization kinetics.^14^

Utilizing the lactation/low Ca rat model we sought to assess the effects of zoledronic acid and sclerostin antibody on matrix maturation during cortical remodeling and the subsequent material properties of the newly formed bone. We used vehicle and NaF treatments as controls. We hypothesized that because of the highly anabolic period following weaning and return to a normal calcium diet, the treatments would not affect bone formation, but would affect matrix maturation.

## Methods

### Experimental design

All animal experiments were performed under institutionally approved protocols. Seventy‐two 10‐week‐old female Sprague–Dawley rats were bred at Envigo (Indianapolis, Indiana) and shipped to Rush University Medical Center (Chicago, IL, USA) on embryonic day 15 (E15). All animals were singly caged, subject to a 12‐hour light/dark cycle, and provided water and food access *ad libitum*. At parturition, litters were normalized to nine pups per dam. During the 23‐day lactation phase, all animals were placed on a low calcium diet (0.01% calcium; Teklab Diets, Madison, WI, USA) to induce substantial resorption.^2^ At weaning, a cohort of eight dams was sacrificed to establish baseline skeletal structure.

The remaining 64 dams were randomized into one of four groups and switched to a normal calcium diet (0.4%) for the recovery phase, which lasted for either 7 or 28 days. The final sample size per group was eight rats per treatment per time, except in the vehicle‐treated group sacrificed at 28 days, where one of the randomly assigned dams was not pregnant. Treatments included saline (vehicle), NaF, zoledronic acid (ZA), or sclerostin antibody (Scl‐Ab). Vehicle, ZA, and Scl‐Ab were provided via weekly s.c. injections. Both ZA and Scl‐Ab were diluted in saline and provided at doses of 1.5 μg/kg, and 5 mg/kg, respectively. NaF was provided in the drinking water at a dose of 100 mg/L. Both ZA and Scl‐Ab doses were specifically chosen to be relatively low, to avoid potential hypocalcemia. The ZA dose was chosen based on previous publications employing low doses in rodents,^15^, ^16^, ^17^ whereas the Scl‐Ab dose used was chosen to mimic the clinical dose used in patients. The dose of NaF was chosen to match that of studies that have demonstrated clear bone remodeling effects.^18^, ^19^

**Figure 1:**
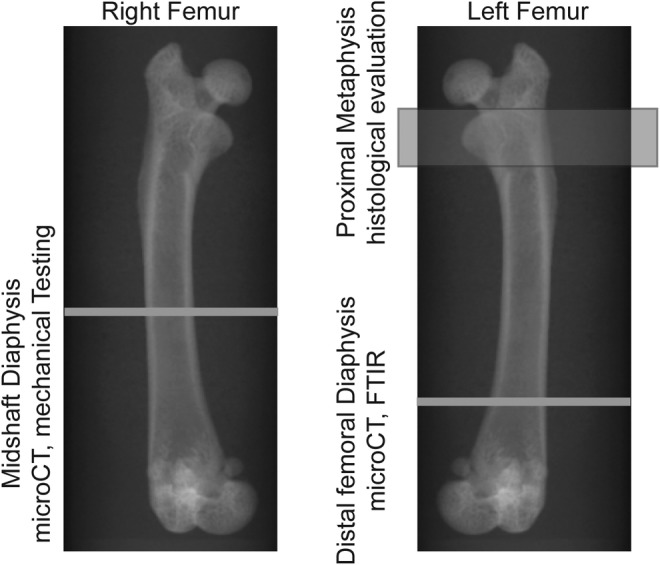
Representative X‐ray images demonstrating the location of various analyses performed as part of the current study. The right femur was used for μCT analysis of the midshaft diaphysis, followed by fracture toughness testing. The distal femoral diaphysis of the left femur was used for μCT and for Fourier transform infrared imaging (FTIR). Finally, the proximal metaphysis from femurs collected in a previously published study using the lactation/low calcium model^1^ was used to evaluate remodeling histologically

At the time of sacrifice, left and right femurs were dissected and cleaned of soft tissues. The left femurs were immediately fixed in 70% ethanol at 4°C for 48 hours. Right femurs were wrapped in saline‐soaked gauze and frozen at −20°C. A graphical representation of the locations of various analyses is presented in Fig.1 and described in more detail in the corresponding sections below.

### Fluorochrome labeling

All animals in each of the four groups received s.c. fluorochrome injections during the recovery phase to allow for tissue‐age‐specific assessment of bone matrix maturation as described previously.^2^, ^14^ Cohort 1 received tetracycline on the day after weaning, calcein on the third day, and Alizarin Red on the fifth day, followed by sacrifice on the seventh day. Cohort 2 received tetracycline on the 8th day, xylenol orange on the 12th day, calcein on the 16th, and Alizarin Red on the 20th day, followed by sacrifice on the 28th day. Using the fluorochrome labels, we planned to isolate bone of specific tissue ages; 2 to 4 and 4 to 6 days in cohort 1 and 8 to 12, 12 to 16, and 16 to 20 days in cohort 2.

### Histological assessment of intracortical and endocortical bone remodeling

To investigate the progression of remodeling in the lactation/low Ca model, we performed an histological analysis at the intracortical and endocortical bone surfaces. Undecalcified femoral bone specimens from our previously published study, in which dams were sacrificed at weaning and at days 1 and 7 during the recovery process, were fixed in 70% ethanol, dehydrated in a graded series of ethanol solutions, and embedded in methylmethacrylate. The embedded specimens, one from each rat at each time point, were cut into 230 to 470 7.5‐μm‐thick serial sections where every 10th section was Masson trichrome‐stained^20^ to assess remodeling activity in the intracortical remodeling events. Prior to staining, sections were deplasticized using 2‐methoxyethyl acetate, followed by dehydration in graded ethanol solutions (99% to 96%) and rehydration in water. Following Masson trichrome staining, sections were treated with a graded ethanol solution (96% to 99%), xylene and mounted with Pertex (HistoLab Products, Gothenburg, Sweden). The abundance of eroded, osteoid, and quiescent surfaces was microscopically assessed in the frontal plane of the left proximal femoral diaphysis under normal and polarized light, rendering the lamellae structure and cement lines more visible. Representative images of the remodeling activities at each time point were obtained using an Olympus UC30 camera mounted on an Olympus BX53 microscope (Olympus, Waltham, MA, USA).

### Bone structure

Bone structure was assessed using μCT (μCT50; Scanco Medical AG, Brüttisellen, Switzerland). The first series of measurements were performed at the distal femoral diaphysis, a site previously identified as a location with significant endocortical and intracortical remodeling in the lactation/low calcium model.^2^ Left femurs were scanned in 70% ethanol at 70 kVp, 57 μA, 1500‐ms integration time, and 2‐μm voxel size. Variables of interest included medullary and total area, cortical thickness, and cortical porosity. The second series of measurements were performed at the midshaft diaphysis of the right femurs to match the location of subsequent mechanical testing. Femurs were scanned in cold saline using the following scan settings: 70 kVp, 57 μA, a 500‐ms integration time, and a 14.8‐μm voxel size. Variables of interest included cortical area, porosity, inner and outer radii, and the moment of inertia.

### Dynamic histomorphometry

After μCT scanning, left femurs were dehydrated in a graded series of alcohol and embedded in an epoxy resin (EpoThin; Buehler). Transverse, cross‐sectional slabs, which were approximately 700‐μm thick, were cut from the embedded femurs at the distal femoral diaphysis region of interest previously scanned by μCT using a diamond‐band saw (E300; EXAKT Technologies, Oklahoma City, OK, USA). Sample slabs were fixed to plastic slides (EpoCure; Buehler, Lake Bluff, IL, USA), followed by grinding to a section thickness of roughly 500 μm and polishing to a mirror surface (Phoenix 4000; Buehler). Dynamic histomorphometry was performed at the endocortical and intracortical surfaces using the fluorochrome labels injected during the bone recovery process (Osteomeasure; OsteoMetrics, Decatur, GA, USA). Mineral apposition rate (MAR) was assessed between each sequential series of fluorochrome labels. Endocortical mineralizing surface per bone surface (MS/BS) was determined as the total endocortical label perimeter divided by the label perimeter of the inner‐most fluorescent label. To determine the intracortical bone surface, the sections were stained with toluidine blue to identify cement lines and the perimeter of remodeled intracortical units were determined by the presence of stained cement lines and polarized lamellae, as described previously.^2^ Bone formation rate per bone surface (BFR/BS) was defined as the multiplication of MS/BS and MAR.^21^

### Fourier transform infrared imaging

High‐resolution reflectance Fourier transform infrared imaging (FTIRI) was performed at the Advanced Light Source (ALS) at the Lawrence Berkeley National Laboratory (Berkeley, CA, USA). Measurements were made between fluorochrome labels using the same polished distal femoral diaphyseal cross‐sections used for dynamic histomorphometry. Matrix compositional measurements were performed at both the endocortical and intracortical remodeling surfaces. Because of limitations in the fluorescent detector, only tetracycline, calcein, and Alizarin Red were clearly identifiable. At the endocortical surface, measurements were made between tetracycline and calcein and calcein and Alizarin. However, at the intracortical surface, Alizarin was not consistently present; therefore, measurements were only made between tetracycline and calcein.

The spectral data were collected using a Nicolet Continuμm IR microscope and a Nicolet 6700 FT‐IR (Thermo Fisher Scientific, Waltham, MA, USA). A total of 256 scans were collected per pixel, with a spectral range of 650 to 4000 cm^−1^, a spectral resolution of 8 cm^−1^, and aperture size of 15 × 15 μm. Background spectra were collected using a gold‐coated slide. The mineral‐to‐matrix and collagen maturity ratios, the carbonate substitution, and mineral crystallinity were obtained following transformation of the reflection spectra into equivalent absorbance spectra and baseline correction according to validated techniques.^22^

Tissue‐age‐specific spectra were integrated to obtain values for the mineral‐to‐matrix and collagen maturity ratios, the carbonate substitution, and mineral crystallinity parameters. The amide I peak was defined by integrating 1600 to 1700 cm^−1^ (baseline 1300 to 1800 cm^−1^), phosphate by 980 to 1200 cm^−1^ (baseline 900 to 1200 cm^−1^), and carbonate by 1414 to 1424 cm^−1^ (baseline 1300 to 1800 cm^−1^). The mineral‐to‐matrix ratio was defined as the phosphate to amide I ratio and carbonate substitution by the carbonate to phosphate ratio. Crystallinity was defined as the ratio of stoichiometric to nonstoichiometric phosphate (1030–1034 to 1019–1023 cm^−1^, baseline 900 to 1200 cm^−1^) and collagen maturity ratio as the ratio of mature to immature collagen crosslinks (1659–1661 to 1689–1691 cm^−1^, baseline 1300 to 1800 cm^−1^).^22^

Tissue‐age‐specific spectra were collected across the cross‐sectional femoral region and averaged to create a single matrix compositional variable at each tissue age for each animal. On average, 15 ROIs were averaged per tissue age, per animal for each endocortical variable and 10 ROIs were averaged for each intracortical variable. For example, the mineral‐to‐matrix ratio measure made within bone of a mean tissue age of 3 days for each animal was obtained by averaging 15 15 × 15 μm ROIs (approximately 3400 μm^2^ of total tissue area) made across the total cortical cross‐section. This averaged value was subsequently used in the statistical analyses described below.

### Fracture toughness

After μCT scanning, right femurs were subjected to controlled notching by first initiating an approximately 0.5‐mm‐deep notch on the posterior face of the diaphyseal midshaft. Subsequent notch sharpening was performed using a custom jig that ensured flat contact of the initiation notch with a fresh razor blade. The notch was deepened to approximately 1 mm, roughly one‐third of the cross‐sectional thickness, using a razor blade irrigated with 1‐μm diamond‐polishing compound (Metadi; Buehler). Lading was performed in three‐point configuration (MTS Criterion) in the anterior‐posterior direction, with the upper loading pin positioned directly above the machined notch, while the outer loading supports were set 15 mm apart. Data was collected using a displacement rate of 0.001 mm/s and collected at 100 Hz.

After loading, the fractured sample was dehydrated via a graded series of alcohol solutions (70% to 100%). The fracture surface was affixed to an aluminum block via double‐sided carbon tape and the exposed surface was carbon‐coated. Scanning electron micrographs (Zeiss, Thornwood, NY, USA) were collected and used to identify the half‐crack angle (Θ). As it was difficult to identify consistent postyield deformation in the samples, the maximum load method was used and the fracture toughness was determined using the initiation angle and the load at failure according to the equation below (Eq. 1):

(1) $$K_{c}=F_{b}\frac{P_{\max}SR_{0}}{\pi \left(R_{0}^{4}-R_{i}^{4}\right)}\sqrt{\pi \theta_{c}}$$

where *K*_*c*_ is fracture toughness, *F*_*b*_ is a geometric factor for thick‐walled pipes described in detail in ref 23,^23^ *P*_*max*_ is the maximum load, *S* is the span length, *R*_*o*_ and *R*_*i*_ are the outer and inner diameter of the cortical diaphysis, and Θ_*c*_ is the half‐crack angle.

### Statistics

Data from the baseline group, sacrificed at the end of the induction period, provide reference values, but were not included in subsequent statistical analyses. Normality was assessed using the Shapiro–Wilk test. Fracture toughness was found to be nonparametric and was log‐transformed for statistical analysis. Two‐way ANOVA tests were performed to assess the effects of treatment, time to sacrifice, and the treatment‐by‐time interaction, on the μCT structural and fracture toughness parameters. When main effects were significant, between‐group differences were compared using a Tukey's test.

FTIRI parameters were averaged per tissue age per animal at both the endocortical and intracortical compartments and treated as independent observations to allow for more detailed description of the maturation kinetics by combining measurements made in cohort 1 (average tissue ages of 3 days and 4 days) and cohort 2 (10 days and 18 days). Dynamic histomorphometry parameters, including the MAR, mineralizing surface per bone surface (MS/BS), and the bone formation rate per bone surface (BFR/BS) were averaged per tissue age per animal and treated as independent events in subsequent statistical analyses. FTIRI and dynamic histomorphometry parameters were assessed using a two‐way ANOVA and compared separately for the endocortical and intracortical compartments. If significant treatment effects or treatment‐by‐time interactions were noted, post hoc analysis was performed using a Tukey's test. A similar statistical approach has been employed in previous FTIR‐based matrix maturation kinetic studies in which separate cohorts of animals were used.^24^, ^25^

## Results

**Figure 2:**
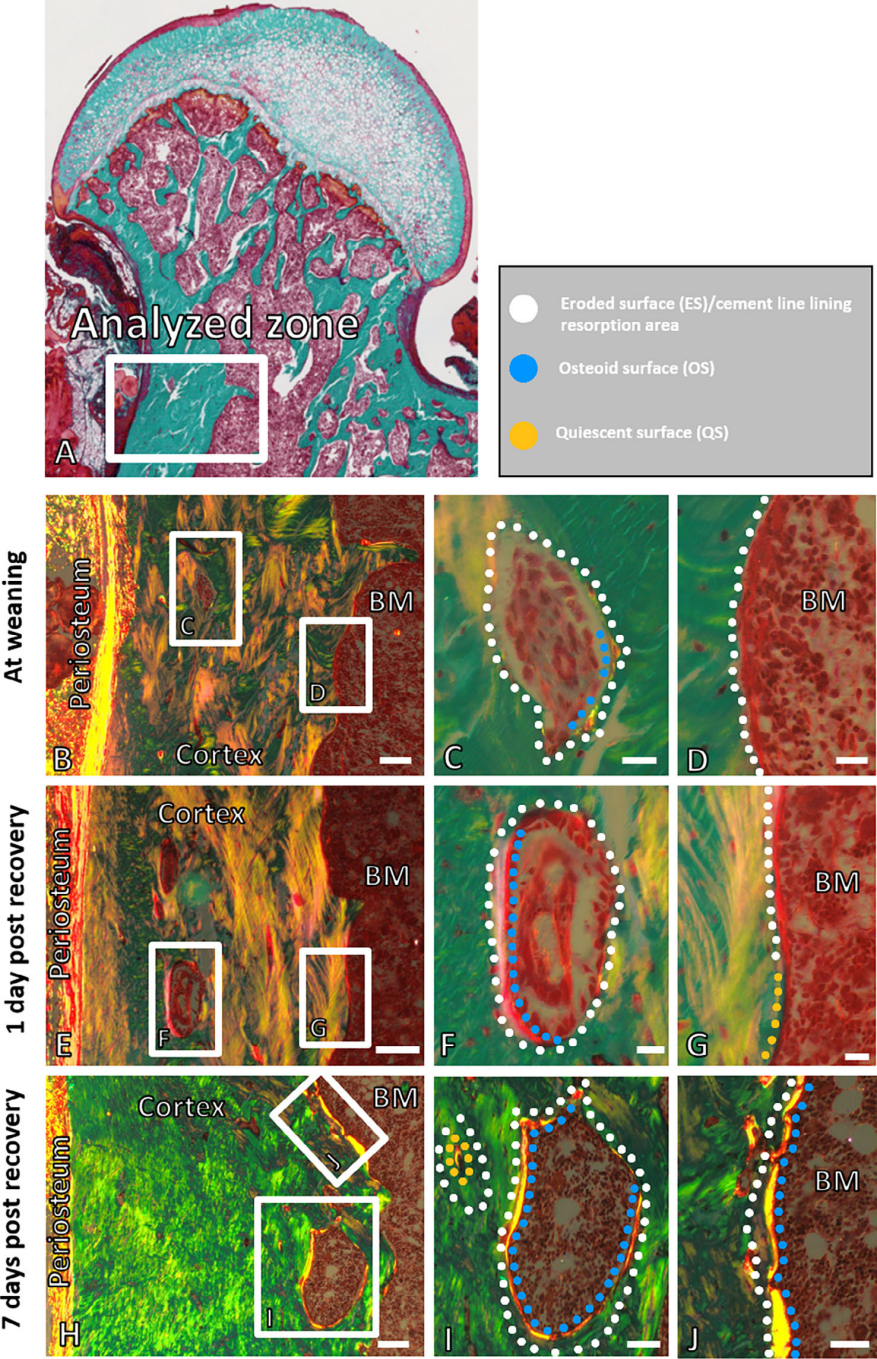
Detailed histologic observations at weaning, day 1, and day 7 of recovery at the proximal femoral diaphysis. The cortex of femoral bone specimens from rats was analyzed over the indicated zone (*A*). The figure illustrates Masson's trichrome‐stained sections (*A*–*J*), subjected to polarized light highlighting the lamella structure and newly formed osteoid (*B*–*J*). Intracortical pores were identified in the proximal femoral diaphysis at weaning, and at day 1 and day 7 of recovery, respectively (*B*–*J*). At weaning, intracortical remodeling canals with eroded surfaces and a low degree of bone formation were observed (*C*), whereas eroded surfaces were observed at the endocortical regions (*B, D*). At 1 and 7 days of recovery, eroded and osteoid surfaces were observed; however, osteoid surfaces predominated the surfaces of the intracortical canals and endocortical regions (*E–J*). Quiescent surfaces were observed at some endocortical regions (*G*) and at some osteons (*I*). Scale bars: *B*, *E*, *H* = 100 μm, *C*, *D*, *F*, *G* = 20 μm, *I*, *J* = 50 μm. BM = bone marrow

Histological evaluation demonstrated a wave of remodeling activities on the intracortical and endocortical bone surfaces (Fig. 2). At the time of weaning, most of the intracortical and endocortical surfaces were characterized as eroded, reflecting an accumulation of remodeling events during the lactation/low Ca diet phase of the experiment, resulting in the nearly exclusive presence of resorption and reversal phases.^26^ Relatively little osteoid surface, reflecting bone‐forming surfaces, was detected at weaning. One day postweaning and return to a normal calcium diet, osteoid surfaces clearly became more abundant at all intracortical and endocortical sites, indicating rapid initiation of bone formation. Seven days postweaning the transition to formative osteoid surfaces on both intracortical and endocortical surfaces was even more apparent. At the sites of formation, the cement lines, reflecting former eroded surfaces, were more deeply buried at 7 days compared with 1 day.

**Figure 3:**
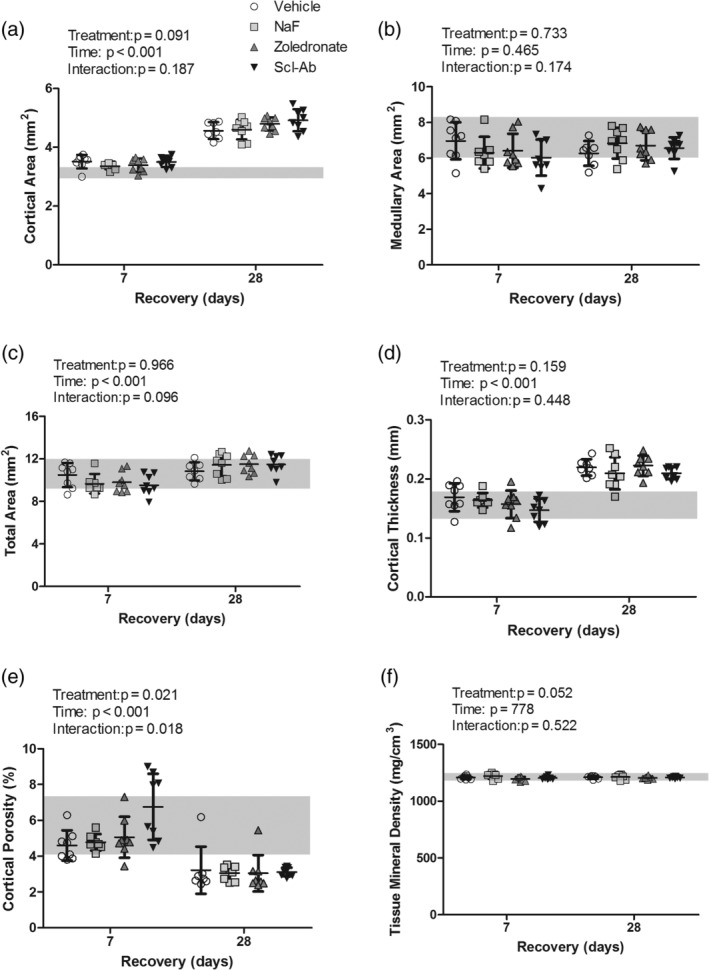
Cortical geometric parameters assessed via high‐resolution μCT at the distal femoral diaphysis. (*A*) Cortical, (*B*) medullary, and (*C*) total area, (*D*) cortical thickness, (*E*) cortical porosity, (*F*) tissue mineral density. Data are presented as the mean (SD) as a function of recovery (time of sacrifice). The mean value and SD for the baseline cohort, sacrificed at the end of lactation, are presented as a solid gray line and the shaded box behind the data bars, respectively. Results from the two‐way ANOVA are presented in the figure legends. The sample size used for statistical comparison was 8, 7, 8, 8 for vehicle, sodium fluoride (NaF), zoledronate, and sclerostin antibody (Scl‐Ab) within cohort 1 and 7, 8, 8, 8 for cohort 2

Consistent with our previous publications in the lactation/low Ca model,^1^, ^2^ high‐resolution μCT analysis at the distal femoral diaphyseal cortical bone showed relatively low cortical area and high cortical porosity levels at the end of weaning (Fig. 3). At days 7 and 28 postweaning, the treatments did not affect cortical area, nor were there any interactions between treatment and time, but there was a significant increase in cortical area over time. Similarly, the total area, medullary area, and cortical thickness showed no treatment effects or interactions. The total area and the cortical thickness had significant time effects, with increasing values over recovery, whereas there was no time effect for the medullary area. Animals treated with Scl‐Ab had a significantly greater cortical porosity when compared with other treatment groups at day 7, which led to significant treatment and interaction effects. However, this increased porosity was reduced to the level of the other treatment groups by day 28. Overall, all treatment groups showed decreasing cortical porosity over recovery, leading to a significant time effect. The cortical tissue mineral density had a nearly significant treatment effect (*p* = 0.052), because of the increased tissue mineral density noted in the NaF‐treated group.

Dynamic histomorphometry was assessed at the same distal femoral diaphyseal site used for high‐resolution μCT analysis. Intracortical MAR was not affected by treatment nor was there an interaction between treatment and time (Table 1). There was, however, a nearly significant time effect attributed to a reduction in MAR over time in all treatment groups. Similar to MAR, intracortical MS/BS and BFR/BS were not affected by treatment nor were there treatment‐by‐time interactions (Supplementary Tables S1 and S2). There were significant time effects, with both intracortical MS/BS and BFR/BS decreasing in all treatment groups during recovery.

**Table 1:** Intracortical Mineral Apposition Rate (MAR)

Intracortical MAR = μm/day				
	Days 1 to 3		Days 8 to 12	
Treatment	Average	SD	Average	SD
Vehicle	2.92	0.66	2.82	1.41
Sodium fluoride	2.93	0.75	2.77	0.81
Zoledronate	3.02	0.50	2.55	0.17
Sclerostin antibody	3.04	0.38	2.26	0.39

There were no treatment (*p* = 0.837) or time (*p* = 0.059) effects, nor was there a treatment‐by‐time interaction (*p* = 0.558).

Endocortical MAR demonstrated a nearly significant treatment effect, a significant time effect, and a significant treatment‐by‐time interaction (Table 2). The earliest measured MAR of NaF‐treated animals (tissue age of 1 to 3 days) was lower than the other treatments, whereas Scl‐Ab‐treated animals showed an increased MAR between 3 to 5 days. The time effects in the endocortical compartment are attributed to a gradual increase in MAR up to a tissue age of 8 to 12 days, followed by a gradual decline in all groups. However, this trend is not consistent across treatments because of the early variation described above, accounting for the significant interaction term.

**Table 2:** Endocortical Mineral Apposition Rate (MAR)

Endocortical MAR= μm/day										
	Days 1 to 3		Days 3to 5		Days 8 to 12		Days 12 to 16		Days 16 to 20	
Treatment	Average	SD	Average	SD	Average	SD	Average	SD	Average	SD
Vehicle	6.45	1.55	5.95	1.21	7.38	0.57	5.51	1.26	4.39	0.98
Sodium fluoride	4.78 a b	0.97	5.71 b	0.86	7.41	2.45	4.95	1.27	4.38	1.15
Zoledronate	5.71	0.72	5.56 b	0.78	7.58	1.28	5.47	0.54	4.30	0.51
Sclerostin antibody	6.24	0.24	8.52 a	2.54	7.50	1.46	4.78	1.04	4.09	1.02

a Indicates significantly different from vehicle measured at the same timepoint.

b Indicates significantly different from sclerostin antibody at the same timepoint.

There was a nearly significant treatment effect (*p* = 0.05) and significant time (*p* < 0.001) and treatment‐by‐time interaction (*p* = 0.006) terms.

Endocortical MS/BS demonstrated a significant time effect and a significant treatment‐by‐time interaction. The interaction term appeared to be driven by suppressed MS/BS in the earliest time points for the NaF and zoledronate groups (Supplementary Table S3). The endocortical BFR/BS parameter demonstrated significant treatment and time effects, as well as a significant treatment‐by‐time interaction because of the suppressed bone formation rates for the NaF‐ and zoledronate‐treated groups in the early time points (Supplementary Table S4).

**Figure 4:**
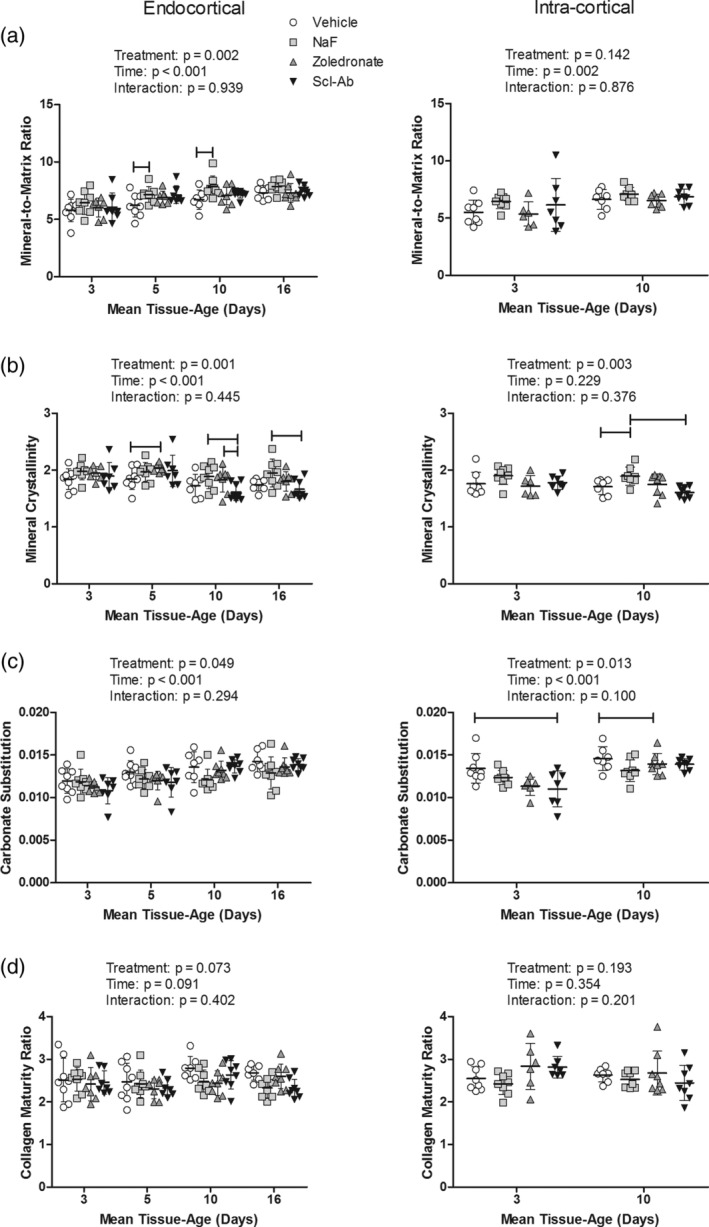
Bone matrix maturation assess via Fourier transform infrared imaging in the distal femoral diaphysis. (*A*) Mineral‐to‐matrix ratio, (*B*) mineral crystallinity, (*C*) carbonate substitution, and (*D*) collagen maturity ratio. Measurements were made in remodeling units within endocortical (left) or intracortical (right) remodeling units. Data is presented as the mean (SD) as a function of tissue‐age. Results from the two‐way ANOVA are presented in the figure legends and between group differences are noted with horizontal bars. The sample size used for statistical comparison was 8, 7, 7, 7 for vehicle, sodium fluoride (NaF), zoledronate, and sclerostin antibody (Scl‐Ab) within cohort 1 and 7, 8, 8, 8 for cohort 2

Treatment significantly impacted the mineral‐to‐matrix ratio at the endocortical compartment, with NaF in particular showing an elevated ratio compared with the other treatment groups (Fig. 4). There was no treatment effect found in the intracortical mineral‐to‐matrix ratio. Both endocortical and intracortical compartments had significant time effects, with an overall increase in the mineral‐to‐matrix ratio with increasing tissue age.

Treatment significantly impacted the mineral crystallinity at both the endocortical and intracortical compartments (Fig. 4). In general, NaF treatment increased the mineral crystallinity, whereas Scl‐Ab decreased crystallinity in the older tissue ages. There were significant time effects for mineral crystallinity measured at the endocortical surface, with an overall decrease in crystallinity as a function of tissue age (Fig. 4). There were no time effects in the intracortical crystallinity.

Treatment significantly impacted the carbonate substitution in both compartments (Fig. 4). In general, all treatments reduced the carbonate substitution compared with the vehicle, although the magnitude of these differences varied with tissue age. The carbonate substitution at both the endocortical and intracortical compartments had significant time effects, with each compartment showing increasing carbonate substitution as a function of tissue age. Finally, there was no significant treatment, time, or interaction for the collagen maturity ratio (Fig. 4).

**Figure 5:**
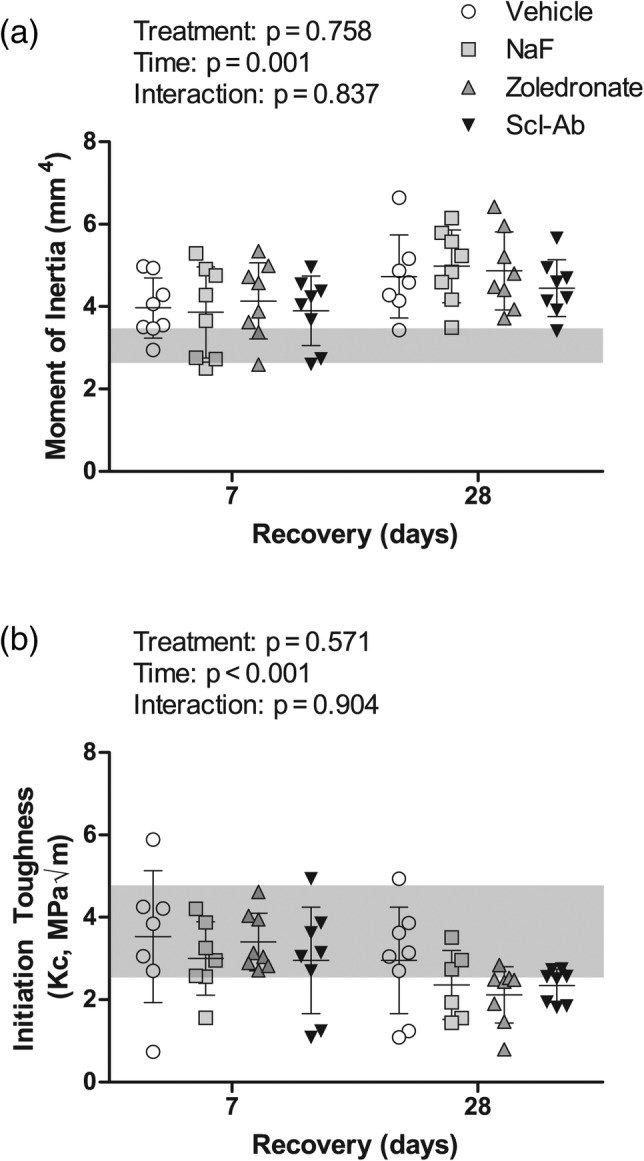
Cortical geometry and mechanical properties of mid‐diaphysis of the femur. (*A*) Moment of inertia in the anterior–posterior direction and (*B*) initiation toughness. Data are presented as the mean (SD) as a function of recovery (time of sacrifice). The mean value and SD for the baseline cohort, sacrificed at the end of lactation, are presented as a solid gray line and the shaded box behind the data bars, respectively. Results from the two‐way ANOVA are presented in the figure legends and between group differences are noted with horizontal bars. The sample size used for statistical comparison was 7, 7, 8, 8 for vehicle, sodium fluoride (NaF), zoledronate, and sclerostin antibody (Scl‐Ab) within cohort 1 and 6, 7, 8, 8 for cohort 2

At the midshaft, treatment did not affect the moment of inertia (Fig. 5). Similar to the cortical parameters within the distal diaphysis, there was a significant time effect, with an increase in the moment of inertia from day 7 to 28. There was no significant treatment effect for *K*_*c*_ (Fig. 5). There was a significant time effect as *K*_*c*_ was consistently lower in bones from animals at day 28 compared with day 7 regardless of treatment. Additional femoral midshaft cortical geometry parameters are presented in Table 3. Of note, is the lack of a time effect in the total area and the outer radius, indicating that the periosteal surface remains largely unchanged, while the medullary area and inner radius decreased from day 7 to day 28 (Table 3).

**Table 3:** Cortical Geometry Measured at the Femoral Midshaft

		Vehicle		NaF		ZA		Scl‐Ab		Two‐way ANOVA		
Variable	Baseline	7 days	28 days	7 days	28‐days	7 days	28‐days	7 days	28 days	Treatment	Time	Interaction
Bone area (mm^2^)	3.26 (0.16)	3.51 (0.31)	4.32 (0.30)	3.29 (0.19)	4.40 (0.28)	3.49 (0.36)	4.44 (0.19)	3.50 (0.22)	4.42 (0.30)	0.576	<0.001	0.517
Total area (mm^2^)	8.35 (0.63)	8.72 (0.61)	8.61 (0.57)	8.33 (0.78)	8.91 (0.63)	8.40 (0.69)	8.87 (0.53)	8.54 (0.55)	8.70 (0.58)	0.996	0.081	0.399
Medullary area (mm^2^)	5.09 (0.58)	5.22 (0.54)	4.29 (0.39)	5.03 (0.66)	4.52 (0.44)	4.91 (0.78)	4.43 (0.53)	5.04 (0.48)	4.28 (0.43)	0.907	<0.001	0.644
Cortical thickness (mm)	0.33 (0.02)	0.35 (0.03)	0.46 (0.02)	0.33 (0.02)	0.46 (0.02)	0.36 (0.05)	0.47 (0.03)	0.34 (0.03)	0.47 (0.02)	0.460	<0.001	0.790
Polar moment of inertia (mm^4^)	7.17 (0.78)	8.01 (1.06)	9.16 (1.20)	7.21 (1.09)	9.771 (1.25)	7.60 (0.95)	9.67 (0.83)	7.81 (0.85)	9.47 (1.19)	0.958	<0.001	0.339
Cortical tissue mineral density (mg/cm^3^)	1224.83 (16.06)	1224.60 (12.36)	1217.77 (29.60)	1220.75 (22.98)	1236.70 (32.20)	1240.55 (26.76)	1224.95 (22.32)	1220.86 (21.36)	1231.20 (16.21)	0.586	0.872	0.217
Cortical porosity (%)	0.5 (0.05)	1.22 (0.55)	0.12 (0.04)	1.60 (0.72)	0.11 (0.03)	0.93 (0.50)	0.19 (0.14)	2.66 (1.34)	0.15 (0.02)	0.003	<0.001	0.002
Inner radius (mm)	1.35 (0.1)	1.37 (0.08)	1.26 (0.06)	1.35 (0.10)	1.28 (0.07)	1.33 (0.10)	1.26 (0.08)	1.34 (0.06)	1.25 (0.07)	0.830	<0.001	0.831
Outer radius (mm)	1.77 (0.07)	1.79 (0.07)	1.79 (0.07)	1.75 (0.09)	1.81 (0.07)	1.77 (0.07)	1.81 (0.05)	1.78 (0.06)	1.80 (0.06)	0.984	0.135	0.594

Data is presented as the means (SD).

Vehicle = Saline; NaF = Sodium fluoride; ZA = zoledronate; Sci‐Ab = sclerostin antibody.

## Discussion

Bone mass is determined by the coordination of osteoclast‐mediated bone resorption and osteoblast‐mediated bone formation. The final phase of remodeling is the maturation of the newly formed bone matrix, which provides bone tissue with its characteristic strength and stiffness. Bone‐targeting treatments are designed to inhibit bone resorption (anticatabolic) or promote bone formation (anabolic), and yet little attention is paid to their effects on matrix maturation. In the current study, we demonstrate the utility of the lactation/low Ca rat as a preclinical model to study the effects of bone‐targeting treatments on matrix maturation independent of their effects on bone remodeling. By timing the treatment specifically to the recovery phase, cortical bone formation occurs in both anticatabolic‐ (ZA‐) and anabolic‐ (Scl‐Ab‐) treated animals at nearly the same mineral apposition rate and amount of forming surface (MS/BS), resulting in similar bone formation rates. At the matrix level, we confirm that NaF significantly increases the mineralization and crystallinity of newly formed bone, zoledronate increases the mineral crystallinity, and Scl‐Ab reduces the crystallinity at older tissue ages and shows evidence of accelerated early mineralization and reduced mineral crystallinity. These findings support the hypothesis that the treatments would not affect bone formation, but would affect matrix maturation. Unexpectedly, despite the treatments affecting matrix maturation, fracture toughness was unaffected.

NaF was used clinically for a brief period to treat osteoporosis because of its positive effects on trabecular bone mass.^27^, ^28^ Despite increased BMD in NaF‐treated patients, large clinical trials demonstrated elevated fracture risk in treated patients.^5^, ^6^, ^29^ One consequence of NaF treatment is the incorporation of fluoride ions into the mineral component of bone and teeth, which increases the mineral crystallinity and mineralization levels.^8^ The current study confirms the elevated mineral crystallinity and mineralization (mineral‐to‐matrix ratio); however, there was no concomitant reduction in fracture toughness. Although osteoporosis patients treated with NaF had increased fracture risk, previous work in growing rodent models failed to find an impact on whole‐bone mechanical properties following NaF treatment.^18^, ^19^ In the current study, we measured cortical fracture toughness to assess the material properties of NaF‐treated bones, but this is not a measure of fracture susceptibility. For example, previous studies have confirmed the mineral crystallinity is more strongly associated with fatigue properties of cortical bone, rather than monotonic properties.^30^ It is also possible that the influence of NaF is felt more strongly at the trabecular‐rich regions, as the most commonly reported sites of skeletal fractures clinically following NaF treatment include the vertebrae and femoral neck.^5^, ^6^, ^28^ Finally, we estimate that less than 25% of the cortical bone at the mid‐diaphysis was formed while NaF was being administered and the majority of this tissue was at the endocortical surface, a surface that provides less mechanical advantage than does the periosteal surface, which appeared to be quiescent. Although there was intracortical remodeling, the total area of this activity was relatively small. Future experiments should be aimed at increasing the amount of newly formed intracortical tissue through multiple reproductive cycles.

Bisphosphonates significantly reduce activation frequency and as a consequence, inhibit bone formation, thereby reducing the amount of bone formed.^31^, ^32^, ^33^, ^34^ Thus, in many animal models the lack of bone formation makes it difficult to study the effects of bisphosphonates on bone matrix maturation. In long‐term human subjects studies, there have been consistent reports of elevated global bone matrix mineralization following bisphosphonate treatment^11^, ^35^, ^36^; however, because formation is suppressed it is difficult to determine whether this is caused by bisphosphonate‐specific effects on the maturation process or by continuation of secondary mineralization in the absence of remodeling.^25^ Using the lactation/low Ca model, we can initiate treatment during the recovery period, thereby specifically concentrating on the effects of bisphosphonates on matrix maturation. In the current study, we focused on zoledronate specifically so that we could compare with the only study in human biopsies that measured matrix composition in bisphosphonate‐treated and untreated bones of similar tissue ages.^9^ Consistent with the findings from Gamsjaeger and colleagues,^9^ we found that in the lactation/low Ca model, zoledronate treatment caused little to no effect on the matrix mineralization, but did increase the mineral crystallinity in newly formed tissue.

Despite the increased mineral crystallinity in response to zoledronate treatment, there was no change in the femoral fracture toughness. Reduced fracture toughness has been previously noted in bisphosphonate‐treated patients that presented with atypical or brittle femoral fractures.^37^ Assuming a sufficient amount of cortical bone had formed during treatment, the current findings suggest that the previously reported degradation in fracture toughness is unlikely a consequence of direct alteration to the matrix maturation process. Similar to NaF, it is also possible that the tissue may be adversely affected in ways that may not be captured by fracture toughness testing. The matrix susceptibility to microcracking, for example, can contribute to fatigue life and therefore fracture risk, but may not be captured in fracture toughness measurements. Indeed, previous research has clearly shown that tissues from bisphosphonate‐treated preclinical animal studies show an increased microcrack burden and reduced fatigue life following cyclic loading^38^, ^39^, ^40^, ^41^, ^42^ and in human biopsy studies have reported extensive microcracking in regions surrounding atypical femoral fractures.^43^ Future work is necessary to determine whether the matrix maturation effects noted in the current study are associated with changes in the fatigue properties of cortical bone. The current study used a relatively low dose and only a single bisphosphonate. As stated above, we focused on zoledronate to allow us to directly compare with the human biopsy findings by Gamsjaeger and colleagues^9^; however, much of the data relating to microdamage and atypical femoral fractures have been reported following exposure to alendronate.^35^, ^37^, ^38^, ^39^, ^42^ Therefore, it is possible that the effects of bisphosphonates on matrix composition and mechanical properties are not universal and may be dependent on the molecular structure of the bisphosphonate used.

Scl‐Ab is a recently approved anabolic agent for osteoporosis. Previous in vivo research has demonstrated that treatment with Scl‐Ab can not only increase bone formation rates, but can also influence skeletal maturation by reducing the mineralization lag time^13^ and accelerate the mineralization kinetics.^14^ These results are consistent with in vitro data that has established that sclerostin protein is an inhibitor of mineralization.^12^, ^13^ In our previous study in nonhuman primates, we found that Scl‐Ab accelerated mineralization kinetics primarily in the intracortical remodeling units.^14^ In the current study, we found evidence of a similar effect at both the endocortical and intracortical compartments, with mineral‐to‐matrix ratio in the earliest tissue age of Scl‐Ab‐treated animals mildly elevated compared with vehicle‐treated controls.

During lactation and reproduction, the bone adapts to shift more of the load‐bearing capacity to the cortical bone^44^ and the majority of the cortical bone lost is on the endocortical surface, which provides the dam with the optimal mechanical strategy.^45^ Therefore, perhaps it is not surprising that the fracture toughness in the baseline and day‐7 groups is comparable to values reported for unaffected female rat femurs.^23^ However, it is surprising that the fracture toughness was reduced at 28 days compared with 7 days. The calculation of fracture toughness (Eq. 1) is partially dependent upon geometric parameters so that the decrease from 7 to 28 days in *R*_*i*_ with no change in the other variables would mathematically lead to lower fracture toughness. Future work is needed to determine whether the fracture toughness parameter recovers to normal levels following a longer period of matrix maturation.

The current study used different cortical regions for the measurement of matrix composition (distal metaphysis) and the mechanical properties (midshaft). We opted to use the distal femoral metaphyseal region for the matrix compositional measurements, as our previous studies had confirmed that there would be sufficient endocortical and intracortical bone remodeling to allow for sufficient sampling of the newly formed tissue.^2^ However, matched anatomical locations would allow for more direct correlation between bone matrix composition and fracture toughness results. Despite this limitation, the study had several important strengths, including a direct comparison of the effects of zoledronic acid and Scl‐Ab, an anticatabolic and an anabolic agent, on bone matrix maturation, which largely confirmed results seen in larger animal models or human studies. Importantly, the current results also demonstrate the potential of using the lactation/low Ca model to evaluate cellular level changes in matrix biology in addition to tissue‐ and organ‐level changes following treatment with osteoporosis drugs. For instance, the model could be used for more mechanistic studies, such as providing an in vivo model to investigate osteoblastic gene expression changes in response to bisphosphonate treatment that have been reported in various in vitro models.^46^

The current study demonstrates the utility of the lactation/low Ca model for the study of cortical bone matrix maturation at the endocortical and intracortical compartments. Despite the differing mechanisms of the bone‐targeting treatments that were tested (anticatabolic versus anabolic), bone structure was restored to the same degree and at nearly the same pace during the recovery period. There were clear treatment effects on the bone matrix maturation process, which were largely consistent with previously reported data, confirming the applicability of the model. Through the use of the lactation/low Ca model, the timing of bone formation can be controlled such that new bone matrix is maturing under the influence of the candidate treatments, allowing for detailed examinations of the effects of matrix maturation and the resulting material properties.

## Disclosure

All authors state that they have no conflicts of interest.

## Supporting information


**Supplementary Table S1** Intracortical Mineralizing surface per bone surface (MS/BS)
**Supplementary Table S2:** Intracortical Bone Formation Rate per Bone Surface (BFR/BS)
**Supplementary Table S3:** Endocortical Mineralizing surface per bone surface (MS/BS)
**Supplementary Table S4:** Endocortical Bone Formation Rate per Bone Surface (BFR/BS)
