# Supplementary material for: Osteoporosis Treatments Affect Bone Matrix Maturation in a Rat Model of Induced Cortical Remodeling
Source: JBMR Plus. 2020 Feb 20;4(4):e10344. doi: 10.1002/jbm4.10344 (PMC7117844; doi:10.1002/jbm4.10344)
Supplement: Supplementary file 1 — Supplementary Table S1 Intracortical Mineralizing surface per bone surface (MS/BS) Supplementary Table S2: Intracortical Bone Formation Rate per Bone Surface (BFR/BS) Supplementary Table S3: Endocortical Mineralizing surface per bone surface (MS/BS) Supplementary Table S4: Endocortical Bone Formation Rate per Bone Surface (BFR/BS) [file JBM4-4-e10344-s001.docx]

**Supplemental Table 1:** Intra-cortical Mineralizing surface per bone surface (MS/BS)

| Intra-cortical Mineralizing surface per bone surface  (MS/BS, %) | | | | |
| --- | --- | --- | --- | --- |
|  | Days 1-3 | | Days 8-12 | |
| Treatment | Average | SD | Average | SD |
| Vehicle | 28.90 | 17.63 | 12.37 | 2.87 |
| Sodium Fluoride | 27.56 | 17.25 | 19.74 | 8.36 |
| Zoledronate | 34.68 | 11.03 | 27.52 | 14.80 |
| Sclerostin Antibody | 30.14 | 12.32 | 21.30 | 12.63 |
| Treatment: p=0.372 Time: p=0.017 Interaction: p=0.849 | | | | |

**Supplemental Table 2:** Intra-cortical Bone Formation Rate per Bone Surface (BFR/BS)

| Intra-cortical Bone Formation Rate per Bone Surface  (BFR/BS, μm^3^/μm^2^/day) | | | | |
| --- | --- | --- | --- | --- |
|  | Days 1-3 | | Days 8-12 | |
| Treatment | Average | SD | Average | SD |
| Vehicle | 89.91 | 62.29 | 35.33 | 21.43 |
| Sodium Fluoride | 74.02 | 37.50 | 52.90 | 24.69 |
| Zoledronate | 91.80 | 63.47 | 68.90 | 35.64 |
| Sclerostin Antibody | 89.02 | 34.29 | 48.83 | 30.62 |
| Treatment: p=0.757 Time: p=0.009 Interaction: p=0.776 | | | | |

**Supplemental Table 3:** Endocortical Mineralizing surface per bone surface (MS/BS)

| Endocortical Mineralizing surface per bone surface (MS/BS, %) | | | | | | | | | | |
| --- | --- | --- | --- | --- | --- | --- | --- | --- | --- | --- |
|  | Days 1-3 | | Days 3-5 | | Days 8-12 | | Days 12-16 | | Days 16-20 | |
| Treatment | Average | SD | Average | SD | Average | SD | Average | SD | Average | SD |
| Vehicle | 95.74 | 3.44 | 76.61 | 15.43 | 99.56 | 2.96 | 98.12 | 3.65 | 92.52 | 3.68 |
| Sodium Fluoride | 90.50*‡ | 5.14 | 79.82‡ | 6.47 | 98.51 | 2.02 | 95.95 | 4.11 | 90.01 | 4.72 |
| Zoledronate | 89.29*‡ | 6.80 | 81.19 | 9.52 | 99.25 | 0.30 | 96.48‡ | 0.93 | 92.06 | 2.81 |
| Sclerostin Antibody | 97.37 | 2.29 | 90.63* | 6.14 | 98.02 | 1.65 | 93.11* | 2.98 | 89.65 | 7.60 |
| Treatment: p=0.165 Time: p<0.001 Interaction: p=0.001 | | | | | | | | | | |
| * indicates significantly different from vehicle measured at the same time-point  ‡ indicates significantly different from sclerostin antibody at the same time-point | | | | | | | | | | |

**Supplemental Table 4:** Endocortical Bone Formation Rate per Bone Surface (BFR/BS)

| Endocortical Bone Formation Rate per Bone Surface (BFR/BS, μm^3^/μm^2^/day) | | | | | | | | | | | | |
| --- | --- | --- | --- | --- | --- | --- | --- | --- | --- | --- | --- | --- |
|  | Days 1-3 | | Days 3-5 | | | Days 8-12 | | | Days 12-16 | | Days 16-20 | |
| Treatment | Average | SD | | Average | SD | | Average | SD | Average | SD | Average | SD |
| Vehicle | 617.82 | 150.92 | | 458.39 | 127.59 | | 736.18 | 74.91 | 544.29 | 146.06 | 406.92 | 96.10 |
| Sodium Fluoride | 433.32*‡ | 96.74 | | 459.43‡ | 102.83 | | 732.67 | 248.59 | 476.19 | 125.32 | 395.24 | 104.06 |
| Zoledronate | 513.05‡ | 95.91 | | 455.24‡ | 107.75 | | 752.50 | 125.74 | 527.82 | 53.76 | 396.30 | 53.25 |
| Sclerostin Antibody | 608.23 | 31.11 | | 772.63* | 241.26 | | 735.51 | 145.51 | 445.60 | 99.19 | 371.10 | 111.74 |
| Treatment: p=0.026 Time: p<0.001 Interaction: p=0.001 | | | | | | | | | | | | |
| * indicates significantly different from vehicle measured at the same time-point  ‡ indicates significantly different from sclerostin antibody at the same time-point | | | | | | | | | | | | |
